# Editorial: Well-being and work motivation brought by technological changes, coping, and adaptations during and post COVID-19 pandemic: Barriers and opportunities

**DOI:** 10.3389/fpsyg.2023.1150726

**Published:** 2023-02-16

**Authors:** Marius Ioan Drugas, Irina Roncaglia, Sebastiaan Rothmann, Stanislava Yordanova Stoyanova

**Affiliations:** ^1^Department of Psychology, University of Oradea, Oradea, Romania; ^2^Education and Children Services, National Autistic Society, London, United Kingdom; ^3^Optentia Research Unit, North West University, Vanderbijlpark, South Africa; ^4^Department of Psychology, South-West University “Neofit Rilski”, Blagoevgrad, Bulgaria

**Keywords:** wellbeing, work motivation, technological changes, coping, COVID-19

It is relevant and important for the field of psychology to establish how technological changes and adaptational strategies during the COVID-19 pandemic reflect on the promotion and sustainability of human wellbeing, and work motivation. The technological changes are aimed at facilitating adaptation during the fluctuating and restrictive circumstances brought by the pandemic and potentially in the post-pandemic society. However, sometimes they may hinder this adaptation, because they require learning and mastering new knowledge and skills to adopt them most effectively and efficiently.

The 14 manuscripts in this Research Topic aim to clarify if the technological changes occurred during and post COVID-19 pandemic have in any ways affected wellbeing and work motivation, and which coping mechanisms have been effective to increase and/or maintain overall wellbeing. There is evidence that COVID-19 pandemic has impacted all spheres of human life, potentially changing our everyday life and challenging welfare. The state regulatory mechanisms imposed remote working, introduced and expanded e-health services as a part of public health policy, and electronic education as social measures to facilitate the fight against transmission of COVID-19. That is why the increased use of technology plays an important role in human life during this pandemic as a mean for implementing, sustaining, and nurturing work tasks, as well as for maintaining social contacts.

The changes brought to human life by COVID-19 pandemic might be perceived differently depending on the individuals' system of reference. The same factors might be perceived as barriers by people intolerant to uncertainty, loss, suffering, frustration, or as opportunities for growth and development by other people. The most common causes of psychological concern during the pandemic included worrying about family, friends, partners, fears of getting and giving the viral infection to someone; frustration and boredom; and changes in normal sleep patterns (Misra et al.). The prolonged pandemic has made numerous people pessimistic, angry, and nurtured anxiety and depression, which have increased the burden on medical staff and hospitals (Tang and Lee). Online medical support has been offered to reduce the risk of infection.

Individuals' mental health and wellbeing have been protected by means of the existing sources of consolation, hope and strength such as human sense of community, closeness, gratitude, and a belief that the pandemic may spur some positive social change (Lossio-Ventura et al.). Even some personality traits usually regarded as negative could be a protective resource. For example it has been established that higher individual employees' narcissism is related to increased felt responsibility for constructive change in organizations, especially in high environmental uncertainty prompted by the COVID-19 pandemic (Lang et al.). Psychological capital that encompasses optimism, self-efficacy, resilience, and hope positively correlates with occupational wellbeing and work engagement (Guo et al.).

Different coping strategies were used to adapt to changes in life imposed by the prevalence of COVID-19 pandemic and by the social measures taken to prevent it. Part of these coping mechanisms are devoted to positively managing and overcoming stress and adapting in different spheres, such as work. It has been found that avoidant coping contributes to teachers' increased burnout and diminished job-related affective wellbeing during COVID19 school closures (Stan). It has been recommended that teachers adopt coping strategies consistent with their personality traits—problem-focused coping and emotionfocused coping for extroverted conscientious teachers; social support focused coping for neurotic teachers (Stan). The teleworkers evaluated higher the coping strategies used by their managers and colleagues than the non-teleworkers (Romeo et al.).

Digitalization and technological changes triggered by the COVID-19 pandemic within short time periods have posed various change demands on employees counteracted by providing organizational change support in different forms (Schlicher et al.). It has been found that behavioral intention to use the new technology correlates positively with organizational change support (instrumental support, informational support, emotional support, and appraisal support), need satisfaction, favorable attitudes toward change, but negatively with frustration and satisfaction with the change process (Schlicher et al.). More change demands (work task changes and work role changes) are related to higher frustration and more unfavorable attitudes toward change (Schlicher et al.).

Technology is a double-edged sword and creates barriers (limited access to Internet, hesitation and distrust of the unfamiliar, isolation, compromises with quality of products and services), as well as opportunities (spending time with family, new skills acquired, providing and seeking help *via* social media, source of information and practices regarding health) for individuals (Misra et al.). The use of technology correlates positively with people's resilience, motivation to work, self-efficacy, and emotional wellbeing during COVID-19 pandemic (Misra et al.).

Perceived severity of coronavirus disease and social influence affected the perception of increased utilitarian and health benefits of technology of mobile payment, which in turn influenced the behavioral intention to use the quick response code mobile payment (Tu et al.).

The elevated level of telecommuting (i.e., working a portion of work hours away from the workplace using technology to conduct work tasks) leads to minimal psychological detachment from work (mentally disconnecting from the work situation), which in turn leads to low wellbeing (low job satisfaction and high emotional exhaustion) that may be improved by family interfering with work (Cheng and Zhang). The teleworkers had a better perception of the organizational measures (internal communication about the situation, management and work organization measures, health and prevention measures, labor and salary measures) taken to deal with the pandemic situation with the exception of the strategies focused on communication with clients, which the non-teleworkers rated more positively (Romeo et al.).

Some age, occupational and personality differences exist in this regard. It has been established studying three different age groups of students (2004, pre-COVID, and post-COVID) that psychological immune capacities of students, i.e., their self-regulation and resilience, seem to decrease through the years (Takács et al.). Students' self-perceived wellbeing (positive attitude toward oneself, others, and student life) contributes to students' openness to learning and to aesthetics, cognitive and behavioral autonomy, intrinsic motivation and identification motivation (Bochiş et al.).

It has been proven the efficacy of integration of three types of knowledge—technological, pedagogical, and content knowledge, for increasing the job-related affective well-being of the teachers (Stan). Teachers should improve their personal technology-related teaching skills to increase their wellbeing in online teaching settings (Stan).

Because of the COVID-19, the occupancy rate of hotel companies has been greatly affected, but high customer engagement (expressed as identification, enthusiasm, attention, absorption and interaction) and positive service evaluation (as service quality, perceived value and customer satisfaction) had positive effects on brand trust of hotel companies and on customer behavioral intention (to re-use a hotel and positive word-of-mouth) (Chen et al.).

Teleworking was more positive evaluated (in relation to technological means; access to information; supervisors' role; time management; productivity, quality and effectiveness, overall satisfaction with teleworking) by the employees in the industrial, distribution and consumption, and service sectors in comparison with the employees in the spheres of education, public administration, and healthcare (Romeo et al.). The attitude of colleagues in teleworking was more negatively evaluated by the employees in the sectors of industry, education, administration, and healthcare in comparison with the employees in the spheres of distribution and consumption, and services (Romeo et al.).

The non-teleworkers were slightly more highly motivated than the teleworkers (Romeo et al.). The most highly motivated teleworkers were those who considered that their managers performed well when coping with the situation and were satisfied with the results they achieved in terms of productivity, quality, and effectiveness (Romeo et al.). The most highly motivated non-teleworkers were those who considered that their managers performed well when coping with the situation (Romeo et al.).

It is necessary to develop managers' competencies in order to develop and maintain relations of trust and support, to foster employees' sense of meaningfulness and responsibility at work in order to keep them motivated (Romeo et al.).

The interventions for improving wellbeing and decreasing burnout should be evidence-based, accessible, promoting knowledge and skill development, facilitating self-awareness, selfregulation, autonomy, collaboration, acceptance, and inclusion of different social groups (Adnan et al.). This has been stated for critical care healthcare professionals, but it is relevant to the other occupational spheres, too.

To conclude, research conducted within this Research Topic was focused on better understanding the relationship between wellbeing and motivation considering the technological changes, as motivational drives direct human behavior to achieve personal, physical, emotional, and social wellbeing, in promoting optimal functioning and adaptations in the face of adversities brought by the COVID-19 pandemic.

## Author contributions

All authors listed have made a substantial, direct, and intellectual contribution to the work and approved it for publication.

